# Emerging role of gut microbiota dysbiosis in neuroinflammation and neurodegeneration

**DOI:** 10.3389/fneur.2023.1149618

**Published:** 2023-05-15

**Authors:** Riddhi Solanki, Anjali Karande, Prathibha Ranganathan

**Affiliations:** Centre for Human Genetics, Bangalore, India

**Keywords:** Alzheimer's disease, neuroinflammation, gut microbiota, blood brain barrier, gut brain microbiota axis, gut dysbiosis, prebiotics

## Abstract

Alzheimer's disease (AD), is a chronic age-related progressive neurodegenerative disorder, characterized by neuroinflammation and extracellular aggregation of Aβ peptide. Alzheimer's affects every 1 in 14 individuals aged 65 years and above. Recent studies suggest that the intestinal microbiota plays a crucial role in modulating neuro-inflammation which in turn influences Aβ deposition. The gut and the brain interact with each other through the nervous system and chemical means via the blood-brain barrier, which is termed the Microbiota Gut Brain Axis (MGBA). It is suggested that the gut microbiota can impact the host's health, and numerous factors, such as nutrition, pharmacological interventions, lifestyle, and geographic location, can alter the gut microbiota composition. Although, the exact relationship between gut dysbiosis and AD is still elusive, several mechanisms have been proposed as drivers of gut dysbiosis and their implications in AD pathology, which include, action of bacteria that produce bacterial amyloids and lipopolysaccharides causing macrophage dysfunction leading to increased gut permeability, hyperimmune activation of inflammatory cytokines (IL-1β, IL-6, IL-8, and NLRP3), impairment of gut- blood brain barrier causing deposition of Aβ in the brain, etc. The study of micro-organisms associated with dysbiosis in AD with the aid of appropriate model organisms has recognized the phyla *Bacteroidetes* and *Firmicutes* which contain organisms of the genus *Escherichia, Lactobacillus, Clostridium*, etc., to contribute significantly to AD pathology. Modulating the gut microbiota by various means, such as the use of prebiotics, probiotics, antibiotics or fecal matter transplantation, is thought to be a potential therapeutic intervention for the treatment of AD. This review aims to summarize our current knowledge on possible mechanisms of gut microbiota dysbiosis, the role of gut brain microbiota axis in neuroinflammation, and the application of novel targeted therapeutic approaches that modulate the gut microbiota in treatment of AD.

## 1. Introduction

Alzheimer's disease (AD) is a chronic age-related progressive neurodegenerative condition characterized by significant memory loss and cognitive impairment that affects around 45 million individuals worldwide. Amyloid beta (Aβ) plaque formation, hyperphosphorylation of tau proteins, and neurofibrillary tangles (NFTs) in brain tissue are pathophysiologic hallmarks of AD (1). These inflammatory lesions result in steady loss of neurons in the sensitive regions of brain ultimately leading to AD. The process of neuroinflammation is associated with the clearance of these deposited aggregates via phagocytosis by microglia. The classical Amyloid hypothesis is based on the assumption that Aβ-42 triggers the structural and functional neurodegenerative process in AD pathology; however, it fails to explain the emergence of other complexities involved in the disorder (2). Due to its complex and multifactorial nature, it is difficult to ascertain the exact cause of AD in each case, however there are certain risk factors associated which include: a. Genetic causes: Mutations in the amyloid precursor protein (*APP*), resulting in two genetically distinct forms, namely familial AD and late-onset or sporadic AD (3). b. Non-genetic causes: Age-related exacerbations, immune system dysfunctions, degradation of anatomical factors, environmental factors, etc.

Given the heterogeneity of Alzheimer's disease and the repeated failure of anti-AD drugs, it is critical to explore new therapeutic targets that modulate disease development and etiology. In recent years, a considerable amount of research has identified that the gut microbiota has a significant influence on regulation of brain function by maintaining the homeostasis between innate and adaptive immunity. This endogenous cross-talk between the gut microbiota and brain is termed as the Microbiota Gut-Brain axis (MGBA). Alterations in the MGBA can significantly influence the progression of neurodegenerative disorders like Alzheimer's by mechanisms such as increased permeability of the gastrointestinal barrier and hyperimmune activation leading to systemic inflammation which can impair the blood-brain barrier, thus, promoting neural injury, neuroinflammation and eventually neurodegeneration (4). An increasing repertoire of experimental and clinical evidence supports gut dysbiosis and gut microbial-host interactions as significant factors in neurodegeneration (5–9). Therefore, the pathogenesis of Alzheimer's disease is facilitated by the complex interplay of gut-derived inflammatory responses, aging, and poor nutrition in the elderly. It is now considered that food-based treatment mainly probiotic supplementation or antibiotic treatment that alter the gut microbiota composition may provide novel preventative and therapeutic alternatives for Alzheimer's disease. This review summarizes the role of gut microbiota dysbiosis in regulation of brain function and its implications in AD supported by animal model studies and available clinical results.

## 2. Gut microbiome and association with diseases

**Figure 1:**
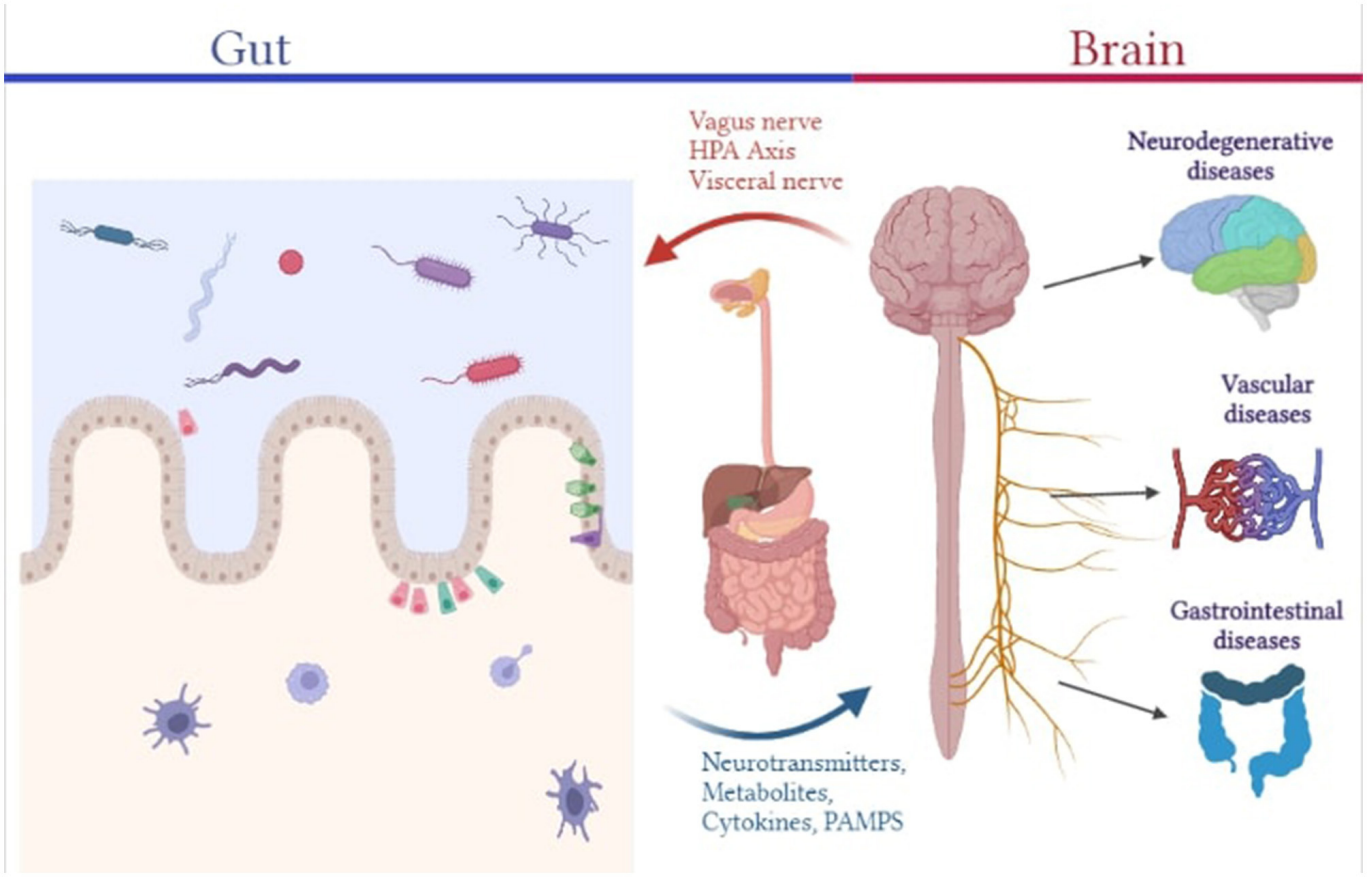
Pictorial representation of the association between the gut microbiome and different human diseases.

Bacteria, archaea, viruses, and eukaryotic organisms are present as resident and transient flora in the body, making up the human microbiome. These micro-organisms have a major influence on our physiology, both in health and diseases. They enable a myriad of functions some of which include, regulation of metabolic activities, protection against potential infections, education of the immune system, etc. and thereby affect many of our physiologic mechanisms either directly or indirectly (10). Characterization of the microbiome of healthy individuals has helped us identify its composition wherein a human adult harbors more than 1,000 species of bacteria belonging to a relatively few known bacterium phyla with *Bacteroidetes* and *Firmicutes* being the dominant phyla (11). The mucus layer and epithelial layer (which contain various junctional protein structures that govern barrier integrity and paracellular permeability) that make up the gut barrier serve as the interface between the outside environment and the host's internal environment. Gut permeability to commensal microbes, microbial derived products (such as metabolites, virulence factors), and other luminal components will increase as gut barrier function is disrupted, contributing to aberrant immune-inflammatory responses like inflammation, allergy, and autoimmune disorder mediated by molecular mimicry and dysregulated T-cell response (4). The functions of such physical and immunological barriers are cross-regulated by interactions between the host and the gut microbiota. T cells play a crucial role in systemic and mucosal immunity effectors. Constant sampling of the intestinal lumen by dendritic cells causes gut bacteria to continuously prime T cells, which predominantly causes regulatory T cell (Treg) proliferation (12). Several commensal microbes, such as *Bacteroides fragilis, Bifidobacterium infantis*, and *Firmicutes*, are capable of inducing the expansion of T_reg_ cells, such as FOXP3-expressing T_reg_ and anti-inflammatory IL-10-producing T_reg_ lymphocytes, which are important in suppressing pathological inflammation caused by aberrant effector T cells, thereby reinforcing gut barrier function (10). Additionally, several microbiota-derived metabolites, such as neurotransmitters and neuromodulators like short-chain fatty acids (SCFAs) produced by *Saccharomyces, Bacillus, Lactobacillus, Escherichia*, and *Bifidobacterium*, biogenic amines (e.g., serotonin, histamine, and dopamine), as well as other amino acid-derived metabolites like serotonin or GABA and tryptophan, have been shown to protect gastrointestinal barrier integrity from the disruptive effects of proinflammatory cytokines caused by an abnormal immune-inflammatory axis. While butyrate is able to stimulate antigen-specific CD8+ T cells, fostering intestinal defense against pathogens, other metabolites affect T cell functionality in several ways, including, apoptotic activation of T cells by ascorbate, suppression of IFN γ and CD8+ T cell growth by mevalonate and activation of Treg cells by glutamate, amongst others. In the brain, microglial activation has been correlated with CD4+ T helper (Th)1 cell levels during the development of AD (13). B lymphocytes, in addition to T cells, are also essential to maintaining CNS immunity and their activation and proliferation can be influenced by microbial metabolites. SCFA and acetate induced IgA antibody production is responsible for maintaining gut barrier composition and integrity (14). A subpopulation of enteric T and B cells may migrate from the gut to the meninges, wherein they affect the local neuro-immune milieu by producing cytokines like IL-17a and IL-10 and IgA class antibodies that interact with central neurons and microglia and prevent meningeal infections (12). Therefore, the enteric nervous system (ENS) is essential as it is capable of regulating the physiology and function of the gastrointestinal tract (GIT) on its own, as well as interact bidirectionally with the CNS via vagal pathways, establishing the gut-brain axis. Maladies in the ENS have been associated with several gastrointestinal disorders (such as IBD, IBS, postoperative ileus), motility disorders (such as constipation), neurodegenerative disorder (such as AD, Parkinson's Disease) and infection-induced gut inflammation (1, 15, 16). Furthermore, *Escherichia coli* and *Bacteroides fragilis* have been shown to potentiate intestinal tumorigenesis in chronic inflammation (17). It has been demonstrated that microbial dysbiosis can cause or even encourage carcinogenesis by producing genotoxins that may alter host DNA. Therefore, prevalent non-pathogenic gut microbiome species serve key functions in occupying the niche and suppressing the growth and colonization of pathogenic organisms that can result in occurrence of infections. With respect to its diverse role, the intestine, is hence referred to as the “second brain” (Figure 1).

## 3. Microbiota gut-brain axis

The bidirectional cross-talk between the brain and gut makes it ideal for maintaining homeostasis across the body, including the gastrointestinal system and the brain through the enteric nervous system (ENS). The ENS is the most extensive element of the autonomic nervous system, with approximately 100 trillion neurons that operate independently of the central nervous system (CNS). Local motor function and blood flow, fluid secretions and transports, and immunological and endocrine activities are all controlled by the neuronal circuits that make up the ENS (18). The vagus nerve links the gut to the brain and spinal cord and is an important part of the microbiota gut-brain axis. The vagus nerve terminates in the nuclei in the brain stem that receive and transmit afferent and efferent fibers. In this way, brain stem nuclei may govern a variety of gastrointestinal processes and transmit signals to other parts of the brain, including the thalamus and cortical areas (18). Several microbial metabolites, including short chain fatty acids and lipopolysaccharides, stimulate the vagus nerve. The presence TLR 2,3,4 and 7 on the surface of vagus nerve also allows them to detect signals from the gut microbes (19, 20). Moreover, it is believed that the vagus nerve functions in Alzheimer's via a mechanism similar to prion disease as a physical transporter of protein aggregates (21). It is implicated in microbial and neurological signaling pathways for satiety, stress, and mood, and hence has consequences for the efficient functioning of both the brain and the stomach (22). Blood circulation can facilitate communication between the gut and the brain. The gut microbiome is also responsible for the production of both pro and anti-inflammatory cytokines that promote the formation of Aβ plaques and neurofibrillary tangles. Microglia are crucial for maintaining tissue homeostasis in the brain and are regarded as intrinsic macrophages of the central nervous system. It is hypothesized that the presence of Aβ is the main trigger for microglial activation in AD. Several studies have shown that Aβ is phagocytosed by activated microglia; nevertheless, these microglia enlarge and eventually lose the ability to process Aβ over time (23–25). AD is marked by microglia dysfunction due to increased activation and synaptic remodeling. SCFAs produced by gut microbiota stimulate microglia to produce neuroprotective IL-10 (12). Other metabolites such as indole and its derivatives have been demonstrated to affect microglial activation and neurotoxicity. The chemical neurotoxic cascade is believed to begin with microglial activation (26). *Peptostreptococcus russellii* that metabolizes tryptophan from the diet into molecules like indole acrylic acid, an indole derivative, act as ligands for Aryl hydrocarbon receptor AhR signaling, has been shown to modulate astrocyte activation, neuroinflammation, and TGF production, impacting microglial activation (27, 28). AhR signaling is an essential component for maintaining intestinal homeostasis as it contributes to the immune response at barrier locations and is influenced by microbial metabolism (29). Recent findings also indicate that peripheral immune system conditioning by the microbiome may cause microglial neuroinflammation. In the CNS, both Aβ plaques and neurofibrillary tangles co-localize with activated glial cells, indicating that gliosis might also play a significant role in AD pathogenesis and neuroinflammation (30, 31). Another microbial metabolite, trimethylamine N-oxide (TMAO) has been associated with the pathophysiology of AD. TMAO is shown to enhance pathologic processes and neurodegeneration by elevating β-secretase activity and exacerbating Aβ buildup (32). Furthermore, elevated levels of circulating bile acids (BAs) produced by bacteria may cause tight junctions to deteriorate, increasing BBB permeability and allowing BAs or peripheral cholesterol to infiltrate the central nervous system (CNS) (33, 34). In the brain, cellular cholesterol results in direct binding to APP, allowing APP to enter the phospholipid monolayers of the lipid membranes where Aβ formation occurs, increasing the synthesis of Aβ (35). The gut microbiome also influences the selection and activity of invariant natural killer cells (iNKT), thereby, serving as an additional link between the gut and brain (36). With respect to gut dysbiosis, recent evidences have shown that the establishment of this endogenous cross-talk between the gut and brain termed as the gut brain microbiota axis is important not only to the normal functioning of the brain but also to the pathogenesis of AD. This is further supported by clinical and pre-clinical studies that have shown that the gut microbiota produces a diverse set of metabolites, all of which regulate the brain activity in one way or another (8, 37–40) (Table 1).

**Table 1:** Gut microbiota and the effects of the metabolites produced on brain functions.

**Sr. No**	**Gut micro-organism**	**Metabolites**	**Effects on brain**	**References**
1.	*Lactobacillus*	Short chain fatty acids (SCFA), Serotonin, Acetylcholine	Increases emotional level, Improves attention, memory and motivation	(41)
2.	*Bifidobacterium*	Gamma-aminobutyric acid	Reduces anxiety, stress, and fear Improves ADHD	(42)
		Tryptophan	Improves behavior relevant to depression	(43)
3.	*Escherichia*	Dopamine, Norepinephrine, Endotoxin and Serotonin	Improves mood, blood flow, sleep regulation, cognition and concentration, hormonal activity	(44–46)
4.	*Saccharomyces*	Norepinephrine	Enhances formation of retrieval of memory	(46)
5.	*Enterococcus*	Histamine, Serotonin	Promotes wakefulness, cognition orchestrates desperate behavior	(44, 47)

**Figure 2:**
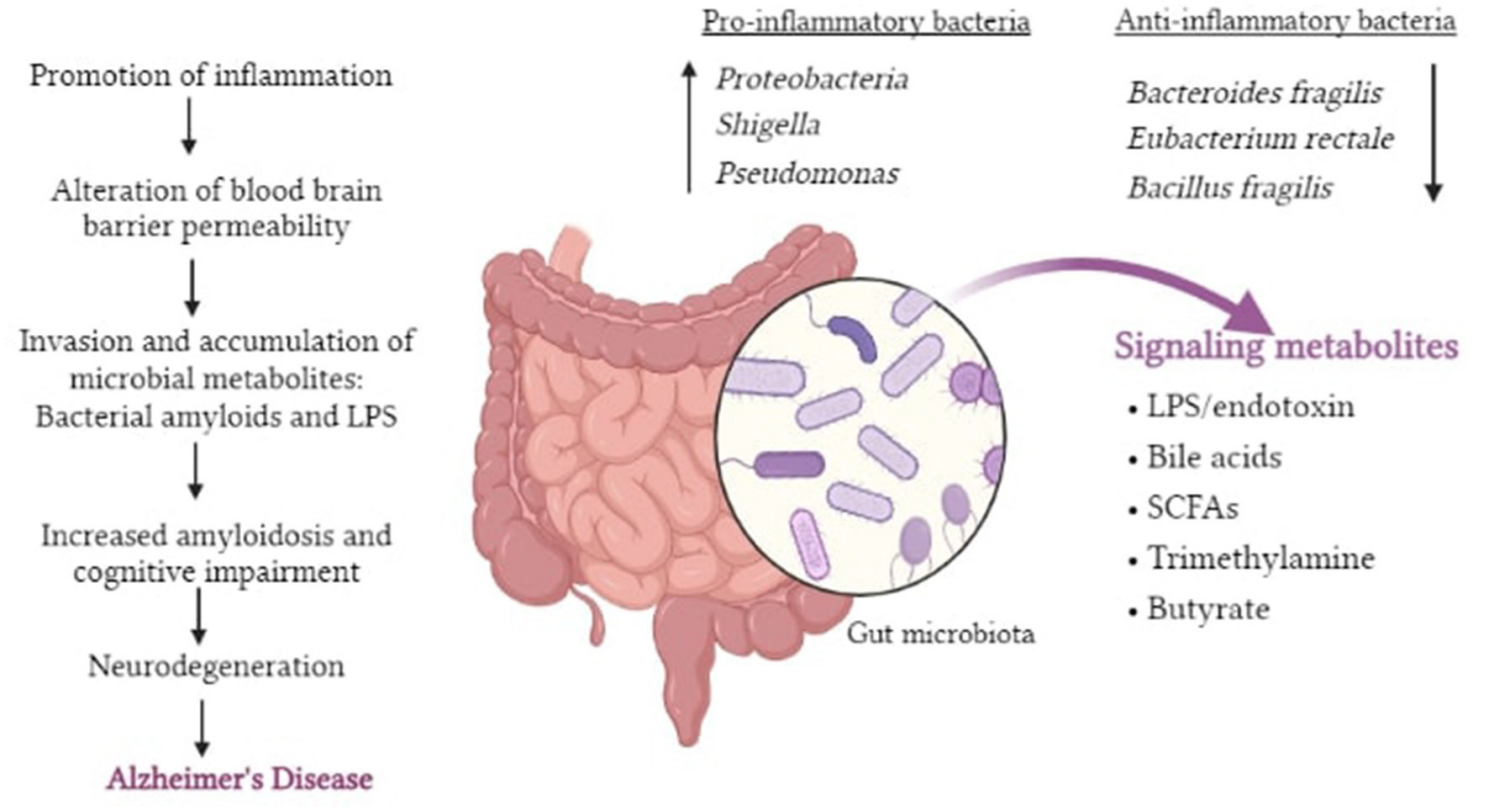
Pictorial representation of potential roles of gut dysbiosis in neurodegeneration and AD.

It is now being suggested that the inclusion of specific microorganisms in the diet, such as probiotics, can be utilized as novel treatment approaches to alleviate neurological diseases. *Bifidobacterium* and *Lactobacillus casei* are two bacteria that have been shown to help with neurological problems (48) (Figure 2).

## 4. Microbiota Gut-brain axis in neuroinflammation

Amyloid plaque formation, a characteristic hallmark of AD pathology is composed primarily of Aβ which is a cleavage product of Amyloid Precursor Protein (*APP*). This transmembrane protein has a role in neural growth, signaling, and intracellular transport, among other biological activities (4). Microglia are the brain's major immune cells, infiltrating the brain early in development and sustaining it in a state of homeostasis as we age. Microglia, the CNS's specialized macrophages, work as phagocytes to eliminate pathogens, cellular debris, and Aβ peptides. Ideally, Aβ peptide-induced acute neuroinflammatory response is a self-limiting, neuroprotective immune response. Aging microglia, on the other hand, are poor at phagocytosing the neurotoxic Aβ plaques seen in AD. Instead, accumulated Aβ plaques activate microglia, resulting in a persistent neuroinflammatory response (2). Interestingly, microglia receive input not only from the brain but also the gut through the vagus nerve and afferent fibers in the vagus nerve send information to the brain about changes in inflammation and proinflammatory cytokine production in the GI tract, influencing neuroinflammation. In a study, it was demonstrated that adult germ-free mice microglia that lack microbial signaling have different densities and morphologies (49). Furthermore, research on critical hypertension found that microglia malfunction and neuroinflammation had an impact on gut microbial populations. Inhibition of microglia activation resulted in alterations in the phylum *Proteobacteria* in this study, suggesting that microglia activity influences gut microbiota composition. Thus, microglia and gut microbiota interact in various ways to maintain the immune system homeostasis which can be disrupted resulting in neuroinflammation (12, 49).

### 4.1. Bacterial amyloids

It is known that accumulation of amyloid plaques is a major characteristic of AD and interestingly, a considerable percentage of amyloids are produced by the human gut flora. Curli, generated by *Escherichia coli*, is the most well-studied bacterial amyloid (50). The synthesis of amyloid proteins aids bacterial cell binding and biofilm formation, as well as resistance to physical and immunological stimuli. Bacterial amyloids may operate as prion proteins through molecular mimicry, resulting in cross-seeding, in which one amyloidogenic protein (curli, tau, Aβ, α-syn, and prion) drives another (e.g., host proteins with a different fundamental structure) to acquire pathogenic β-sheet structure (4). A new term “Mapranosis” which is Microbiota associated proteopathy and neuroinflammation is now being proposed to describe the impact of microbial amyloids in the body (51). Many organisms of the human microbiome, including *Streptococcus, Staphylococcus, Salmonella, Mycobacteria, Klebsiella, Citrobacter*, and *Bacillus* species, have recently been demonstrated to be capable of forming extracellular amyloids. Further, they can also trigger the innate immune system as pathogen associated molecular pattern (PAMPS) with a response consisting of TLR4 (52), TLR1 and TLR2, NFκB, iNOS, and pro-inflammatory micro RNAs (5, 53). The microbiota may have an impact on the inflammatory reactions to amyloid deposits in the brain, and this may influence cerebral damage. As a result, gut exposure to bacterial amyloid proteins may prime the immune system, amplifying immune response to endogenous neuronal amyloid formation in the brain (54).

### 4.2. Lipopolysaccharides

**Figure 3:**
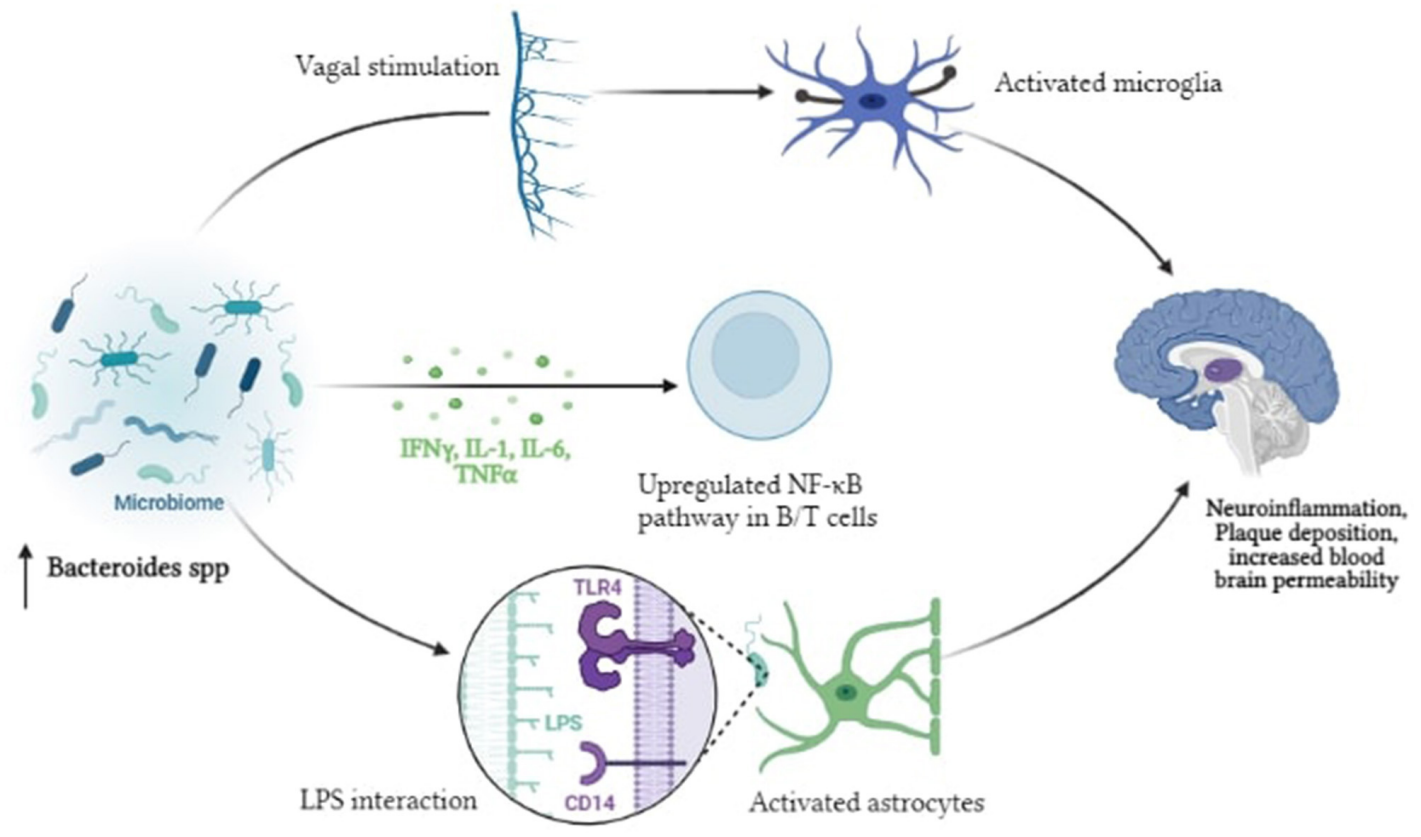
Pictorial representation of the proposed mechanism of LPS driven neuroinflammation in AD.

According to the endotoxin hypothesis of neurodegeneration, endotoxin, or Lipopolysaccharide (LPS), which is present in the outer membrane of all Gram-negative bacteria, penetrates the blood-brain barrier to cause neuroinflammation and neurodegeneration (55). The proposed pathway is initiated by the dysbiosis of gut microbiota that results in enrichment of Gram-negative bacteria in the gut microbiome and is associated with LPS derived inflammation (56). Several *in vivo* and *in vitro* experiments have revealed that LPS activates several intracellular molecules that alter the expression of many inflammatory mediators, thereby contributing to or initiating neurodegenerative progression (57). Peripheral LPS may be involved in the development of Aβ plaques and neuroinflammation in the brain. The proinflammatory cytokines TNF-, IL-1β, and IL-6 which have the ability to reach the periphery, are produced when LPS stimulates the enteric nervous system (58). They function by disrupting the blood-brain barrier, triggering inflammatory pathways, activating TLR4, astrocytes, and microglial cells in the gut (59). This in turn, triggers NFκB activation, increasing cytokine production and Aβ deposition *Bacteroides fragilis, Enterobacteriaceae* and *Lactobacillus johnsonii*, recognized LPS-producing gut bacteria, are thought to play a role in inflammatory signaling in AD patients via the NFκB pathway (60) (Figure 3).

### 4.3. Gut inflammation and gut barrier dysfunction

The mucus layer, intestinal epithelium, and lamina propria make up the intestinal barrier. When this barrier is disrupted, permeability increases, allowing pathogens and their potentially toxic by-products to enter the circulation (a process called as atopobiosis) (61). The gut microbiome is enriched by the presence of many bacteria that strive to maintain the integrity of the gut barrier. For example, the mucin degrading bacteria *Akkermansia muciniphila* enhances gut barrier function while also lowering obesity and systemic inflammation, whereas, *Lactobacillus plantarum, E. coli Nissle*, and *Bifidobacterium infantis* are probiotic bacteria that improve the intestinal barrier by enhancing the production of proteins that create tight junctions (62, 63). In the event of mucosal architectural disruption, an intestinal inflammatory process leads polymorphonuclear cells to migrate from the circulation to the gut mucosa or even farther to the gut lumen. Researchers have now found a way to indirectly measure this process of intestinal inflammation by assessing the concentration of a protein called Calprotectin in stool samples. This small calcium-binding protein (a heterodimer), accounts for 60% of neutrophil cytosol protein content and possesses antibacterial effects (64). Calprotectin levels in the cerebrospinal fluid (CSF) and in the brain of Alzheimer's patients are much higher, promoting amyloid aggregation and co-aggregation with Aβ. According to one study, higher fecal calprotectin levels were identified in nearly 70% of AD patients, suggesting that it might enter circulation and contribute to neuroinflammation (65). It is hypothesized that the presence of such calcium binding proteins in the gut or directly in the brain may lead to the production of amyloid fibrils. Increased intestinal permeability (“leaky gut”) as a result of gut inflammation and dysbiosis associated with gut barrier dysfunction may contribute to the process of neurodegeneration (4, 66).

## 5. Triggering factors of dysbiosis

In recent years, many theories have been proposed to explain the potential triggers that result in gut microbiota dysbiosis which include, direct microbial action of microbes, indirect actions such as antimicrobial protection hypothesis and hygiene hypothesis, processes related to aging in patients, etc. (18).

### 5.1. Direct microbial induced in AD

The evidence that the gut microbiota is the possible cause of AD pathophysiology is provided primarily from studies in model organisms. It is now increasingly observed that, in humans, bacterial [for example, *Borrelia burgdorferi* and *Chlamydia pneumoniae* (67, 68)] and viral [for example, Cytomegalovirus (69)] and Herpes simplex virus type 1 (70) infections may be one of the initiating factors of AD. Chronic *Helicobacter pylori* infection in AD patients has been known to trigger the release of inflammatory cytokines. It was also shown in neuroblastoma cells that exposure to *H. pylori* filtrate causes tau hyperphosphorylation similar to that seen in AD tau pathology (65). All of the bacteria can function cooperatively to increase infectious load in the brain of AD patients. An increase in the pro-inflammatory bacteria *Escherichia* and *Shigella* and a decrease in the anti-inflammatory bacteria such as *Eubacterium rectale* in plasma of patients with cognitive impairment and brain amyloidosis, are linked to elevated levels of IL-1β, CXCL2, and NLRP3 (38). Dysbiosis-induced cognitive impairments that promote AD can manifest in a variety of ways. To begin with, as aforementioned, these bacteria account for changes in the concentrations of particular neurotransmitters. Furthermore, several investigations have found that the gut microbiome can affect synaptic plasticity proteins and receptors such as NMDA receptors, brain-derived neurotrophic factor (BDNF), and serotonin receptors (71). In healthy individuals, the gut microbiota functions to produce neuroprotective compounds such as fatty acids and antioxidants and hence, any detour from the normal state can contribute significantly to the neuroinflammation.

### 5.2. Age-induced dysbiosis in AD

**Figure 4:**
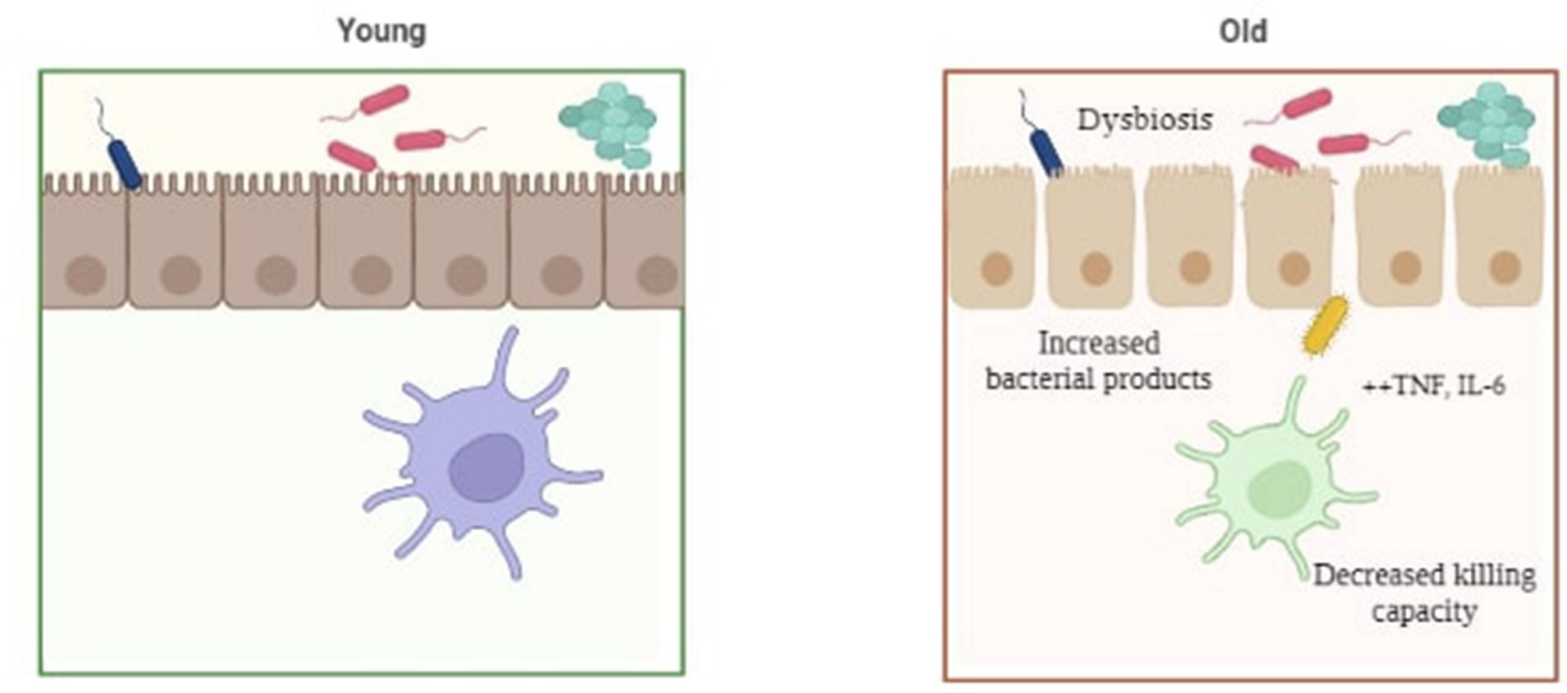
Pictorial representation of gut dysbiosis in young v/s elderly individuals.

Alzheimer's is a disease that is associated with increasing age, mainly 65 years and above. Preclinical and experimental evidences linking the gut microbiota and AD have proposed a theory termed as “age-related dysbiosis”, which hypothesizes that AD might possibly develop through the immune system's aging process (15 and 60). Age-related elevations in circulation and tissue levels of pro-inflammatory cytokines including tumor necrosis factor (TNF) and IL6 have been observed in both humans and mice (72). Thevaranjan et al. (73) also illustrated that age-related microbial dysbiosis can cause an increase in gut permeability and inflammation with aging. In fact, it has been demonstrated that the composition of the gut microbiota varies with age, with an increase in *Proteobacteria* and a decrease in probiotic bacteria such as *Bifidobacteria*, and neuroprotective compounds, such as small chain fatty acids (SCFAs) observed with increased age (74). Furthermore, in healthy aged adults, a correlation has been shown between the loss of microbiome function, especially genes that encode SCFAs, and increasing levels of circulating proinflammatory cytokines. It has been described that age-related dysbiosis and neurological deterioration are related, with the former mediating chronic low-grade inflammation as a common basis for a wide range of age-related abnormalities, or what is now increasingly recognized as “inflamm-aging” (75) (Figure 4).

### 5.3. Hygiene hypothesis of AD

Similar to the process of education of cells of the adaptive and innate immunity, wherein they are taught to differentiate between self and non-self in order to destroy a foreign antigen, the immune system must be sensitized to microbes in order to mount an effective immune response. Insufficient exposure to microorganisms has been linked to immunological dysregulation in AD. According to the “hygiene hypothesis,” many characteristics of contemporary living (such as excessive use of antibiotics, diet habits, sanitation, clean drinking water, etc.) are linked to reduced rates of exposure to microorganisms such as commensal microbiota that are otherwise important (76). Low microbial encounter can in turn lead to low lymphocyte turnover leading to immune dysregulation. Low-level chronic immune system stimulation causes naive T-cells to adopt a suppressive regulatory phenotype that is required for the control of both type-1 and type-2 inflammation. The proliferation of regulatory T-cells (T_reg_) may be inhibited in those patients who do not get enough immunological activation (77). AD has been defined as a disease of systemic inflammation, with elevated type-1 dominant inflammation in the AD brain and periphery: a probable marker of T_reg_ insufficiency. Immune dysregulation as a result of insufficient immunological activation may increase the risk of AD via the T-cell system (78). These findings support the hygiene hypothesis by emphasizing the relevance of immune cell components in the development of AD. Furthermore, in the presence of viral infections or dietary regimens toxic to gut flora, patients bearing genes for AD familiar forms, such as apolipoprotein E (ApoE)-4 allele carriers, have a greater chance of AD conversion. Overall, it is believed that the hygiene hypothesis for Alzheimer's disease (HHAD) predicts that AD incidence is positively associated to hygiene.

## 6. Experimental evidences

The evidence for the role of dysbiosis of gut microorganisms in AD pathogenesis has so far mostly come from animal models. The most established model organisms for studying AD pathology include conventionally raised AD (CONVR-APPPS1) mice models (40), Drosophila models (79), Zebrafish models (80), among others along with clinical data. When the gut microbiota of traditionally grown APPS1 mice and wild type mice is compared, there is a considerable increase in the concentration of *Bacteroidetes* as the age of the mice increases (1, 3.5, and 8 months old) followed by sharp decrease in the number of *Cyanobacteria, Firmicutes* and *Proteobacteria* (40). Similar kinds of results are also inferred using human samples where fecal microbiota compositions of AD and non-AD patients were examined in an investigation. Reduced microbial diversity, decreased quantity of *Firmicutes* and *Bifidobacterium*, and increased abundance of *Bacteroidetes* were found in the feces of Alzheimer's patients (Vogt et al., 217). The increase in CSF fluid biomarkers of AD pathology was associated to a rise in relative bacterial abundance (81). Another set of experiments commonly involve the use of germ-free (GF) mice (without any gut microbiome) in comparison with transgenic APPPS1 mice. GF mice (GF-APPS1) presented a significant reduction in Aβ pathology when compared to normally grown APPS-1 transgenic mice. This is further supported by histopathological examinations which showed reduction in cerebral Aβ load in GF mice as opposed to CONVR-APPS1 mice when stained with Thioflavin T dye (40). Furthermore, 5xFAD mice (a transgenic model of AD; expressing human APP and PSEN1 (Presenilin-1) transgenes with a total of five AD-linked mutations: APP KM670/671NL, APP I716V, APP V7171, and PSEN1M146L and PSEN1L286V) showed increased amyloid-protein precursor (APP) accumulation in the gut and Firmicutes/Bacteroidetes ratio (8). These findings imply that changes in the gut microbiota in animal models is linked to Aβ pathology in the brain (8). Another interesting model system being used to enhance our understanding of gut microbiome dysbiosis and its implications in AD is Drosophila. Induction of enteric dysbiosis by oral infection of non-pathogenic *Enterobacteria* (Ecc15), can significantly colonize the gastro-intestinal tract of adult flies ectopically expressing Aβ-42 in their brains. Surprisingly, as compared to controls without infection, the brains of infected amyloid transgenic flies showed a considerable increase in vacuolar degeneration. These evidences showed that greater neuronal loss in the transgenic brain was caused by intestinal infection which was further confirmed employing a reliable apoptotic marker caspase 3 where increased apoptosis was observed in flies infected with Ecc15 (79). With respect to humans, in individuals with cognitive impairment and brain amyloidosis, higher abundance of proinflammatory *Escherichia/Shigella* and decreased abundance of anti-inflammatory *Eubacterium rectale* were related with peripheral inflammation, according to a recent study (38). All of the aforementioned results indicate a strong correlation between alterations in the gut microbiome, presumably due to an increase or decrease in specific microorganisms or the production of cerebral markers, and AD pathogenesis. Thus, the impact of gut microbiota on brain function is consistently being researched, and the mechanisms of the brain-gut-microbiota axis that contribute to neuroinflammation and neurodegenerative disorders are being highlighted (Table 2).

**Table 2:** Recent data on the role of microbiota in AD pathogenesis from animal and human studies.

**AD model**	**Main findings**	**References**
1. CONVR-APPPS1 and GF mice	Change in the gut microbiota composition: A significant reduction in cerebral Aβ pathology in GF mice compared to control mice; colonization of GF mice with microbiota from conventionally-raised mice increased cerebral Aβ pathology, whereas colonization with microbiota from WT mice was less effective in increasing cerebral Aβ levels	(40)
2.5X FAD mice	Changes in the composition of the feces microbiota with increasing age; human APP expressed not only in the brain but also in the gut tissue and lowered trypsin level in fecal proteins	(8)
3. APOE^−/−^ mice	*Porphyromonas gingivalis* active invasion and infection-induced complement activation in APOE^−^/^−^ mice brains	(37)
4. AD rat model	*Lactobacillus plantarum* improved cognitive function by restoring acetylcholine levels, reducing Aβ plaque formation	(82)
5. Transgenic Drosophila	*Enterobacteria* infection promotes immunological haemocyte recruitment to the brain, which exacerbates the development of AD; elimination of haemocytes reduces neuroinflammation and prevents neurodegeneration	(79)
6. Living AD subjects	Increased pro-inflammatory and decreased anti-inflammatory bacteria. LPS levels were significantly greater in AD plasma specimens, and there was a positive association between level of blood monocyte/macrophage activation in the disease groups	(38).
7. Post mortem brain samples	Poole et al. (37) reported the presence of LPS from *Porphyromonas gingivalis* in sections of AD brains. 16S rRNA sequencing revealed increased bacterial populations in AD brain tissue (39). DNA specific to *Borrelia burgdorferi* was found in senile plaques by Miklossy	(83)

## 7. Potential therapeutic interventions for AD

The gut microbiota–brain axis is still a focus for future Alzheimer treatments. Immunotherapy and treatment that targets the gut microbiota are two amongst the multiple potential therapeutic strategies for Alzheimer's disease. Anti-Aβ and anti-tau target antibodies, Aβ vaccinations, and cytokine inhibition are all immunotherapy targets. Moreover, if the gut microbiota plays a role in AD, certain drugs that can change its composition, such as antibiotics or diet-based interventions can have a positive or negative impact on the disease. Prebiotic/probiotic supplementation and fecal microbiota transplantation are possible gut microbiota target therapies for restoring a diversified, healthy microenvironment (84, 85).

### 7.1. Antibiotic perturbations in AD

Antibiotics are commonly used to suppress or prevent bacterial colonization in the body. As a result, broad-spectrum antibiotics can have a significant impact on the gut microbiota's composition, biodiversity, and colonization for a long time after administration. According to numerous studies, several antibiotic treatments cause short and/or long-term alterations in the gut microbiota in both humans and other models (86–89). Furthermore, both preclinical and clinical research have shown that antibiotic intake and the resulting dysbiosis are correlated to changes in behavior and brain chemistry. Minter et al. (88), have demonstrated that administration of a cocktail of antibiotics termed as ABX [composed of gentamicin (1 mg/ml), vancomycin (0.5 mg/ml), metronidazole (2 mg/ml), neomycin (0.5 mg/ml), ampicillin (1 mg/ml), kanamycin (3 mg/ml), colistin (6,000 U/ml), and cefoperazone (1 mg/ml)] in APPS1 transgenic mice causes a shift in the gut microbial composition that is confirmed with 16SrRNA sequencing method. In addition to this, the effect of ABX therapy on Aβ deposition in the mice brain was investigated by immunostaining brain sections with the Aβ-specific 3D6 antibody34. In these findings, male ABX-treated APPS1 mice showed a substantial 2.27-fold reduction in combined cortical and hippocampus Aβ plaque load as compared to vehicle controls. Consequently, in these mice, there is an increase in neuroinflammatory state and cytokine levels that promotes AD (88). Antibiotics that disrupt the equilibrium of gut flora, such as streptozotocin and ampicillin, are among the most harmful. Antibiotics like as streptozotocin have been used to produce sporadic AD forms in animal models with impacts on learning and memory skills, contributing to this idea (89). Interestingly, in contrast, it is also established that antibiotics can help people with AD through a variety of ways, including prevention of neuroinflammation. This is seen with rapamycin, which is the natural inhibitor of the mammalian enzyme target of rapamycin (mTOR) in addition to possessing antiaging characteristics. Upregulation of the mTOR signaling system is linked to major pathogenic processes in Alzheimer's disease. mTOR inhibitors, such as rapamycin, have been shown to improve AD-like pathology and cognitive deficiencies in a variety of animal models, highlighting their therapeutic potential (90). Nonetheless, the use of antibiotics presents as an attractive option to treat AD and other neurodegenerative conditions, however it should be carefully considered for humans as the insurgence of antibiotic resistance might negate any potential advantages it entails.

### 7.2. Probiotics, prebiotics, and AD

**Figure 5:**
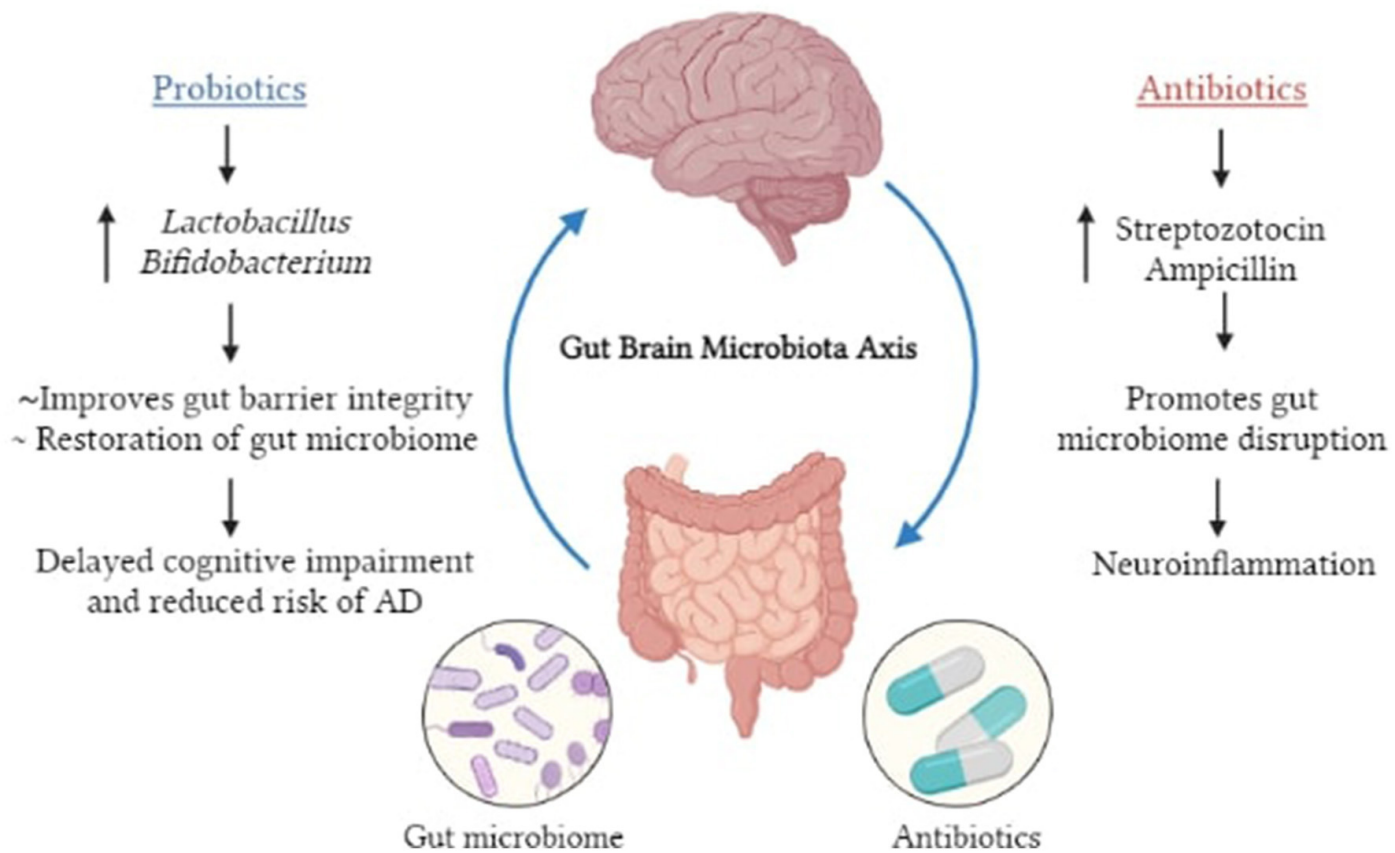
Pictorial representation of the influence of antibiotics and probiotics on MGBA.

Probiotics are microorganisms that benefit the host's general health, whereas prebiotics are substances (mainly fiber) that serve as food for these bacteria. Probiotic supplementation, probiotic enhanced foods, dietary fiber intake, and foods rich in prebiotics are all examples of diet-based therapies. Bacterio-toxins (such as bacteriocins) can be secreted and produced by probiotics, which can inhibit bacterial invasion and pathogen attachment to epithelial cells (91). They also improve barrier integrity and mucus formation by having a trophic impact on the intestinal mucosa as well as effects on epithelial cell cytokine release. Kaur et al. (92) administered probiotic formulation VSL#3 for 8 weeks to a 6-month-old APP^NL − G−F^ (*Lactobacillus plantarum, Lactobacillus delbrueckii subsp. Bulgaricus, Lactobacillus paracasei, Lactobacillus acidophilus, Bifidobacterium breve, Bifidobacterium longum, Bifidobacterium infantis*, and *Streptococcus salivarius subsp. Thermophilus*). They found that acetate, lactate, butyrate, isobutyrate, and propionate levels were elevated in the serum after VSL#3 changed the composition of the gut microbiome, increasing the concentration of*, Lachnospiracea, Clostridia*, and *Akkermansia*. According to a meta-analysis in 2019, owing to their anti-inflammatory and antioxidative properties, probiotics may enhance the cognitive function in AD and MCI patients (93). In a preclinical study on AD mice, Bonfili et al. (94), observed that the treatment of SLAB51 mixture (containing *Streptococcus thermophilus, Lactobacilli*, and *Bifidobacteria*) could minimize Aβ burden, improve cortical atrophy, and restore the defective ubiquitin proteolytic system and autophagy. Moreover, in the brains of 5xFAD transgenic mice, *Lactobacillus plantarum* C29 was demonstrated to modulate microglia activation, decrease Aβ deposition and limit NF-κB activation (95). Transgenic AD mice treated with probiotics are shown to have greater cognitive function and a decreased level of Aβ plaques in the hippocampus when compared to untreated AD mice (63). With respect to clinical studies, in a randomized, double-blind, placebo controlled multicenter trial, Kim et al. (96, 97) observed that probiotics modify the gut microbiome while also promoting mental flexibility and reducing stress in healthy older adults suggesting they can be used for therapeutic purposes. Akbari et al. (62) conducted a similar study comparing the results of giving probiotic milk (including *Lactobacillus acidophilus, Lactobacillus casei, Bifidobacterium bifidum*, and *Lactobacillus fermentum*) vs. control milk (200 ml/day) to AD patients for a 12-week period. They discovered that individuals who received probiotic treatment saw a marked improvement in their cognitive score on the Mini Mental State Examination (MMSE) along with reduced oxidative stress (malondialdehyde) and inflammation (C-reactive protein) markers. These findings suggest that consumption of a nutrition diet rich in probiotics and prebiotics, as well as other nutrients, delays neurocognitive impairment and minimizes the risk of AD. The observed effects maybe attributable to restoration of the gut microbiome and other contrasting actions of AD associated pathologies such as insulin resistance. Oral bacteriotherapy using probiotics has recently been effective in treating and preventing many diseases. Despite the presence of many promising candidates such as *Lactobacillus* and *Bifidobacterium*, with respect to AD, the effect of probiotics as a plausible treatment remains largely unexplored as additional evidence is required to design a probiotic formulation that is effective and reliable. Due to the absence of stronger evidence-based proof of their ability to promote health and of any negative effects, major medical regulatory bodies have not approved any probiotic formulation as a treatment approach. Moreover, it has been noted that probiotic consumption may result in major adverse outcomes like sepsis, particularly in populations like the elderly, immunocompromised and critically ill patients (98). Therefore, the administration of probiotics and prebiotics can be a step toward personalized medicine as the effectiveness of this mode of treatment relies entirely on the dosage required, the proportion of each bacterial strain used and most importantly the severity of the disease that will differ from patient to patient (99) (Figure 5).

### 7.3. Fecal microbiota transplantation: bugs as drugs?

**Figure 6:**
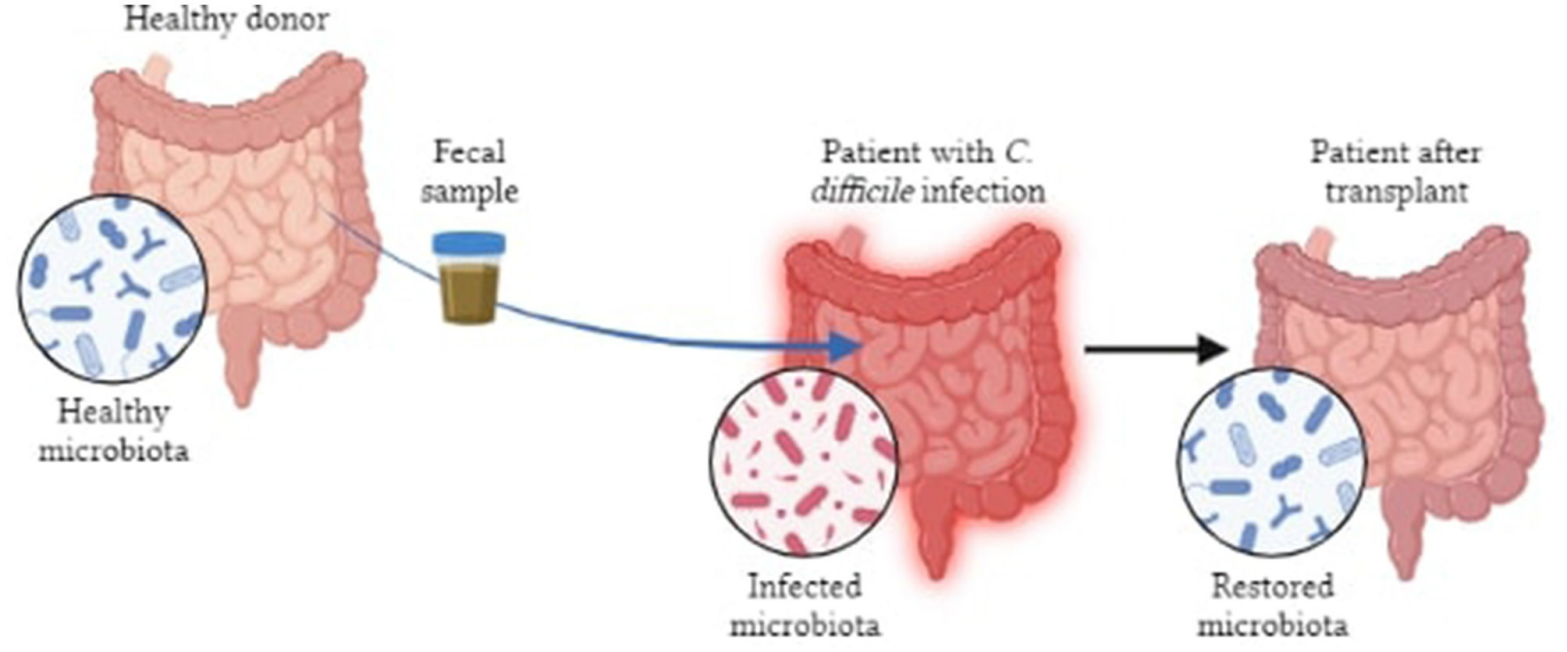
Pictorial representation of FMT in patients with bowel infection.

FMT (fecal microbiota transplantation) is being investigated as a possible treatment where gut microbiome is correlated to pathophysiology of several conditions (100). FMT is the procedure of introducing pre-screened feces into patients' GI tracts in order to restore function and diversify their GM. FMT has the ability to reverse some dysbiosis, engraft bacteria that promote health, while briefly exposing the recipient to beneficial microbes. The gut microbiota of AD mice when transplanted enhances amyloid deposition, demonstrating that the gut does affect AD pathophysiology (101). Sun et al. (102) and colleagues have shown that, in APP/PS1 mice, FMT therapy improved cognitive impairments and reduced Aβ deposition in the brain which was accompanied by phosphorylation of tau protein and decreased levels of Aβ40 and Aβ42. Another study indicated that use of FMT could alleviate AD pathology in mouse models (103). At present, FMT is being used to treat *Clostridium difficile* infections whereas trials related to cancer, IBD and neurodegenerative diseases are under way (102). However, it is difficult to rationalize timing and dose regimens because of the incomplete mechanistic and biological understanding of FMTs, while the possibility of reversing the dysbiosis has not yet been fully established (104). Exposing the immune system of patients to allogenic strains of FMT may be harmful if they have disorders like IBD or autoimmune conditions that can result in adverse pathologic immune responses. Walter et al. (105), have also highlighted the safety concerns (both short and long term) pertaining to donor-recipient compatibility following personalized transplants which include the transmission of communicable diseases, unintended predisposition to chronic non-communicable diseases and possible risks of antibiotic pre-treatments, amongst others. Even though, FMT is showing promising results in preclinical studies, its application to humans is limited by standardization, route of administration, safety assessment, patient acceptance and tolerability, etc., (85) (Figure 6).

## 8. Discussion

So far, we have summarized how dysbiosis may damage the GIT, its associated disorders, as well as its contribution to neurodegeneration, that occurs via the bidirectional crosstalk between the gut and brain called the Microbiota Gut-Brain axis (4). The gut microbiota, which produces amyloid, LPS, and other toxins, may play a role in systemic inflammation and the destruction of physiological barriers. Bacteria or their products can travel from the gastrointestinal tract and oronasal cavity to the CNS, which is particularly dangerous in the elderly (51). Bacterial amyloids may function as seed proteins for prion proteins, causing misfolding and increasing native amyloid aggregation. Furthermore, gut microbiota by-products may stimulate microglia, increasing the inflammatory response in the CNS, leading to pathologic microglial activity, increased neurotoxicity, and decreased amyloid clearance (12, 49). Infectious or sterile inflammatory stimuli may promote Aβ production through TLRs, given Aβ's function as an antimicrobial peptide. The microbiome determines AD diseases and neuroinflammation, according to emerging research, and AD pathogenesis modifies gut microbiota composition. Immune cell maturation, SCFA, LPS, and cytokine production, gut epithelial and BBB permeability, and gut microbiota diversity are all influenced by the MGBA (15, 55).

In the development of AD, this complex system involves a variety of processes that operate synergistically. While the biology of AD is complex, understanding key pathways and processes involved in progression is critical for developing novel treatment options. Our knowledge on the function of gut microbiota in AD and other neurodegenerative disorders has hitherto been based on preclinical or cross-sectional human trials (8, 37, 38, 40, 83, 92). The role of MGBA in AD is now being studied primarily through compositional microbiome data obtained from 16S rRNA marker gene sequencing (8, 39, 88). While this methodology has set the groundwork for research, uncovering the complex range of pathways involved in the MGBA would need an integrated transcriptomics, metagenomics, and metabolomics approach. The minimal number of human studies acts as a limiting factor in determining the directionality of reported alterations in the gut microbiota in AD, such as if gut microbiome dysbiosis is caused by a change in food, medicine, or stress in AD patients. This problem is circumvented by the advent of various AD based model organisms like mice, rat, drosophila, etc. that greatly enhanced our understanding of not only the dynamics of the MGBA but also provided a range of potential therapeutic strategies, for example, antibiotics, probiotics, fecal matter transplantation, etc. that can be explored for treatment purposes. The idea of employing antibiotics along with dietary regimens composed of probiotics that act cooperatively in a cost-effective manner is being clinically tested as a therapeutic, or preventive intervention for AD. Thus, multi-omics investigations in animal models, carefully selected human cohorts, and *in vitro* mechanistic studies will reveal the underlying processes of the gut microbiota–brain axis and its influence on Alzheimer's disease, dementia, and other neurological disorders. In concert with the significance of the microbiome, interestingly, the role of the human mycobiome, and virome is also now being researched extensively in order to connect the dots between the pathophysiology of not only neurological disorders but also several malignancies and other diseases (106). Despite the fact that there are several options now available as therapeutics in AD apart from anti-AD drugs, the inclusion of pro-, pre-, or antibiotics in actual clinical practice and personalized medicine still requires further studies to understand the specific effects of these therapeutics in the alleviation of AD.

## Author contributions

This article has been written by RS with the help of AK. All authors contributed to the article and approved the submitted version.
